# Association between Expression of Insulin-like Growth Factor-1 (IGF-1), IGF-1 Receptor (IGF-1R), and Hypertension-Mediated Organ Damage (HMOD) Parameters in Leukocytes and Plasma of Children/Adolescents with Primary Hypertension

**DOI:** 10.3390/jpm14030255

**Published:** 2024-02-28

**Authors:** Renata Grzywa-Czuba, Joanna Beata Trojanek, Jacek Michałkiewicz, Izabela Kubiszewska, Łukasz Obrycki, Aldona Wierzbicka-Rucińska, Mieczysław Litwin

**Affiliations:** 1 Department of Microbiology and Clinical Immunology, The Children’s Memorial Health Institute, Al. Dzieci Polskich 20, 04-730 Warsaw, Poland; j.trojanek@ipczd.pl (J.B.T.); j.michalkiewicz@ipczd.pl (J.M.); 2 Department of Immunology, Nicolaus Copernicus University Collegium Medicum, M. Sklodowskiej-Curie 9, 85-094 Bydgoszcz, Poland; i.kubiszewska@cm.umk.pl; 3 Department of Nephrology, Kidney Transplantation and Hypertension, The Children’s Memorial Health Institute, Al. Dzieci Polskich 20, 04-730 Warsaw, Poland; l.obrycki@ipczd.pl (Ł.O.); m.litwin@ipczd.pl (M.L.); 4 Department of Biochemistry & Experimental Medicine, The Children’s Memorial Health Institute, Al. Dzieci Polskich 20, 04-730 Warsaw, Poland; a.wierzbicka@ipczd.pl

**Keywords:** primary hypertension, children, insulin-like growth factor-1, IGF-1 receptor

## Abstract

A decrease in IGF-1 is often linked to inflammation. Low systemic and local IGF-1 production and downregulation of IGF-1R expression may precede and predict PH development in children/adolescents. Leukocyte mRNA expression of IGF-1 and its receptor (IGF-1R) and plasma IGF-1 were measured in a group of 39 PH children/adolescents (29 boys and 10 girls) and 35 age-matched normotensive children (19 boys and 16 girls) using the RT-PCR and ELISA tests. The expression of the IGF-1R protein was assessed by flow cytometry. Plasma IGF-1 concentration was evaluated with ELISA. The expression of IGF-1 and IGF-1R and plasma concentrations of IGF-1 did not differ between groups. However, the PH children had a decreased percentage in IGF-1R-bearing lymphocytes (*p* = 0.02) and monocytes (*p* = 0.0003), as well as a low density of IGF-R in monocytes (*p* = 0.02). The IGF-1 expression was negatively correlated with pulse-wave velocity (PWV) (r = −0.49), systolic blood pressure (SBP) (−0.44), and carotid intima-media thickness (cIMT) (−0.43). The IGF-1R expression was negatively correlated with PWV (r = −0.42) and SBP (r = −0.41). Our results suggest that early subclinical hypertensive arterial injury is associated with lower activity of IGF-1-IGF-1R expression and loss of protective actions.

## 1. Introduction

Primary hypertension (PH) is an etiologically complex syndrome. There is a hypothesis called the Page mosaic theory. The theory explains that at least three critical systems (excluding genetic and environmental factors), such as the endocrine, nervous, and immune systems, have affected the regulation of arterial blood pressure [1].

The factors such as IGF-1 (insulin-like growth factor-1) and the IGF-1 receptor represent the molecules of these systems. It is essential to understand these parameters’ relationships to the PH’s pathomechanism.

Insulin-like growth factor 1 (IGF-1) is an insulin homolog protein mainly produced in the liver under the control of the growth hormone [2,3]. IGF-1 is produced locally by many tissues, including vascular smooth muscle cells and endothelial and immune cells such as monocytes, macrophages, and lymphocytes [2]. The IGF-1 receptor (IGF-1R) is a transmembrane tyrosine kinase in virtually every mammalian cell, including VSMCs and endothelial and immune cells. Ligand binding to IGF-1R activates the intrinsic tyrosine kinases phosphorylation and subsequent induction of IGF-1/IGF-1R signaling pathways [2]. IGF-1/IGF-1R interaction in endothelial and VSMCs mediates angio-protective effects. Locally produced IGF-1 supports blood vessel reparation and downregulates inflammation-related oxidative stress [2]. IGF-1 participates in the regulation of regional blood flow and blood pressure via stimulation of endothelial nitric oxide synthase (eNOS) activity and nitric oxide (NO) production in endothelial cells [4] that results in the induction of vasodilation and increased blood flow [5]. Genetic IGF-1 deficiency in mouse models leads to elevated blood pressure, supporting the role of IGF-1 in blood flow and pressure regulation [6]. Serum IGF-1 levels are high in hypertensive adult patients, possibly because of the induction of several adaptive responses, such as ventricular hypertrophy and vascular remodeling [7] Human studies on the role of IGF-1 in hypertension are limited, and results conflicted. Some studies showed higher IGF-1 levels in hypertensive patients compared to normotensive subjects [6,8,9]. Other studies suggest a neutral relationship between blood pressure and IGF-1 levels [2,10]. Hypertension is also related to low vascular relaxation response to IGF-1. Spontaneously hypertensive rats (SHRs) had reduced IGF-1-mediated vasodilation in the aorta, and IGF-1 did not mediate regulatory effects on renal hemodynamics [11]. These observations suggest that the reduced vascular response to IGF-1 may be essential in developing arterial hypertension. It has been suggested that increased matrix metalloproteinase (MMP) activity in hypertension results in the proteolytic cleavage of the extracellular IGF-1R alfa subunit [11]. More recent, extensive cross-sectional studies reported a significant inverse correlation between blood pressure and IGF-1 [12], later confirmed in extensive prospective studies in a group of hypertensive female subjects not diagnosed with diabetes [10]. Few studies have also found an association between high IGF-1 levels and left ventricular hypertrophy in hypertensive patients, a significant predictor of the severity of cardiovascular disease. Insulin-like growth factor-1 (IGF-1) can induce myocardial hypertrophy independently of insulin, and insulin can stimulate cardiomyocyte growth by interacting with IGF-1 receptors due to its structural similarity to IGF-1 [5,7]. All these data indicate the critical role of the IGF-1/IGF-1R axis in PH development both in the experimental hypertension models and the adult human subjects. However, there are no data on IGF-1/IGF-1R expression in PH children/adolescents, presenting early stages of hypertension development in humans.

Here, we studied the relationship between leukocyte IGF-1/IGF-1R mRNA, IGF-1R protein expression, plasma IGF-1 levels, and hypertension-mediated organ damage (HMOD) parameters in pediatric PH patients.

## 2. Materials and Methods

### 2.1. Patients

The project was of a prospective and cognitive nature. The studies were conducted according to the Declaration of Helsinki Guideline on Good Clinical Practice and were approved by the local Bioethics Committee (No. 44/KBE/2016). Patients and their parents or legal guardians consented to participate in the study.

Ultimately, a population of 74 was qualified for the project. Recruitment occurred at the Department of Nephrology, Kidney Transplantation and Hypertension of the Children’s Memorial Health Institute in Warsaw.

The study patients group consisted of 29 boys and 10 girls with newly diagnosed primary hypertension (median age 15.93). The control group was healthy normotensive adolescents, with a median age of 15.2 (19 boys and 16 girls). The exclusion criteria were any chronic infectious diseases or other non-infectious diseases with skeletal and vascular malformations, acute infectious diseases six weeks before recruitment, secondary arterial hypertension, and antihypertensive treatment. Arterial hypertension was diagnosed according to the national recommendations of the Polish Society of Hypertension of 2018 [12] and pediatric guidelines of the European Society of Hypertension of 2016 [13]. In all PH patients, arterial hypertension was confirmed by 24 h ambulatory blood pressure monitoring (ABPM) using the oscillometric device (SpaceLabs Monitor 90207, Spacelabs Healthcare, Snoqualmie, WA, USA). All children underwent a full protocol of assessment of HMOD and biochemical diagnostic laboratory tests.

### 2.2. Medical Examinations

The evaluation of the parameters of NTP-related cardiovascular damage:

1. Mass of the left ventricle using the Aloca Prosound Alpha-7 (Hitachi Healthcare Americas, Lexington, MA, USA) ultrasound machine and the cardiological probe; the left ventricular mass index (LVMi) was calculated, the incorrect value of which determines the diagnosis of left ventricular hypertrophy;
2. The thickness of the carotid intima-media thickness (cIMT) complex and the wall cross-sectional area (WCSA) using the Aloca Prosound Alpha 7 and a linear probe;
3. Elasticity of large arteries based on pulse-wave velocity (PWV) using the oscillometric method using the Vicorder device (SMT Medical, 80 Beats Medical, Berlin, Germany);
4. Pulse-wave analysis (PWA) and evaluation of central systolic arterial pressure were assessed with an oscillometric method using the Vicorder device (SMT Medical).

The above methods were described in more detail previously [14].

### 2.3. Basic Biochemical Diagnostics

Biochemical laboratory tests (glucose, insulin, uric acid, lipid profile, liver profile, and creatinine) were performed for all patients, and in the control group, only those necessary for the implementation of the project (fasting glucose, insulin measurements, and lipid profile).

### 2.4. RNA Isolation and Real-Time PCR

Total RNA was isolated from peripheral leukocytes using the method recommended by the manufacturer (NucleoSpin RNA Blood, Macherey-Nagel kit, cat. 740200.500, Germany, Switzerland, France, and the USA). RNA sample concentration and purity/integrity (260 nm and 280 nm absorbance) were assessed using the spectrophotometric technique (NanoDrop2000c, Thermo Fisher Scientific, Wilmington, DE, USA). One microgram of total RNA for each sample was converted into cDNA in the reverse transcription–polymerase chain reaction (RT-PCR) using the TaqMan Reverse Transcription Reagents kit (Thermo Fisher Scientific, cat. N8080234, USA) and a Veriti thermal cycler. Measurement of mRNA expression for the genes IGF-1, IGF-1R, and the reference gene glyceraldehydes-3-phosphate dehydrogenase (G3PDH) were performed by real-time PCR using the SYBR Green PCR Master Mix buffer and 10 nmol for each of the forward and reverse primers (Thermo Fischer Scientific, cat. 4304970; sequences: IGF-1: F: AGG AAG TAC ATT TGA AGA ACG CAA GT, R: CCT GCG GTG GCA TGT CA; IGF-1R: F: CGC AAC GAC TAT CAG CAG CT, R: AGA TGA GCA GGA TGT GGA GGT; G3PDH F: GCG GGG CTC TCC AGA ACA TCA T; R: CCA GCC CCA GCG TCA AAG GTG), using a ViiA 7 Real-Time System thermal cycler (Thermo Fisher Scientific, ViiA ^TM^ 7, Carlsbad, CA, USA) according to the manufacturer’s recommendations. Analysis of each sample was measured in duplicate. Gene expression in the study group was presented as a relative value in relation to the expression level in the control group after normalization to the expression level of the G3PDH reference gene in both groups according to Pfaffl’s mathematical formula [15].

### 2.5. Immunoassays

Peripheral blood was collected on EDTA and centrifuged to obtain plasma (1500 rpm, 10 min). Hemolyzed and lipemic samples were avoided as they influence the final result. The measurement of the concentration of insulin-like growth factor-1 (IGF-1) in the plasma was performed by quantitative enzyme immunoassay ELISA sandwich type according to the instructions of the purchased commercial IGF Human ELISA kit (IGF-1, ELISA kit, ref. E20 Mediagnost, Reutlingen, Germany) with analytical sensitivity: 0.09 μg/L. Reference ranges for human IGF-1 by age and gender are provided in the assay. The samples were appropriately diluted 1:21 prior to testing. The particular dilution buffer neutralized the function of IGF-1 binding proteins (IGFBPs). Thus, it was possible to measure the concentration of free IGF-1 in the plasma. Absorbance was measured at 450 nm using an ELx800™ Absorbance Microplate Reader (BioTek Instruments Inc., Winooski, VT, USA) and GEN5 analysis software (BioTek, Instruments Inc., Base Code: 3.07, Winooski, VT, USA). IGF-1 concentration was automatically calculated by the program based on the standard curve. The sample dilution (21-fold) was used to estimate the final result. Results were obtained in ng/mL.

### 2.6. Assessment of Leukocyte Membrane IGF-1 Receptor Expression by Flow Cytometry

**Figure 1:**
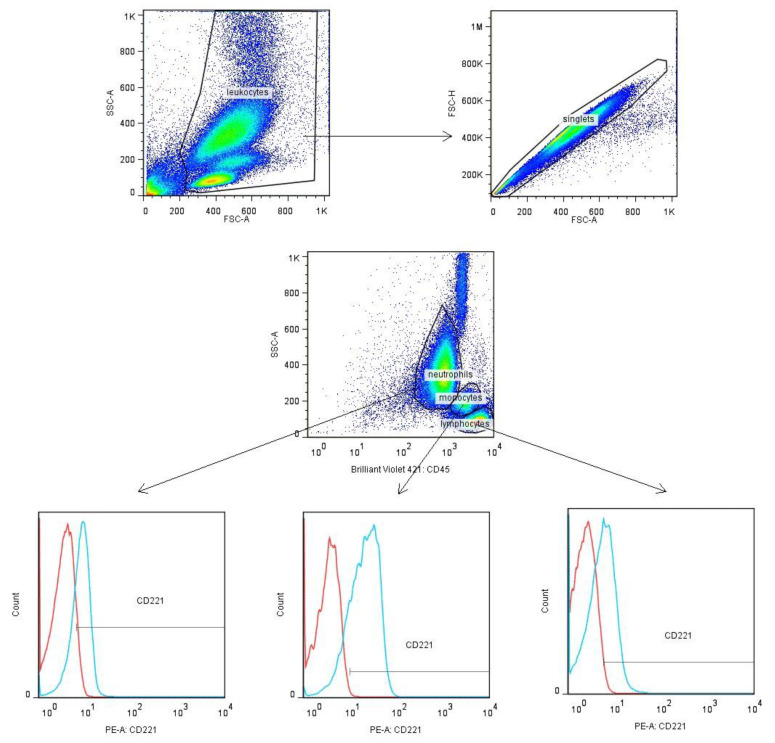
Gating strategy used to identify populations of peripheral blood leukocytes expressing CD221/IGF-1R by flow cytometry (red lines—isotype control; blue lines—protein expression).

A 100 μL of EDTA blood each was added to two flow cytometry tubes (test and control tube) and mixed with monoclonal antibodies (mAbs) anti-CD45 (clone HI30) conjugated with Brilliant Violet 421 (BV421) (BD Pharmingen^TM^, San Diego, CA, USA). Additionally, mAbs anti-CD221/IGF-1R (clone 1H7), conjugated with phycoerythrin (PE) (BD Pharmingen, San Diego, CA, USA), were added to the test tube and isotype-matched antibodies that lack specificity to the target conjugated with PE to the control tube (isotype control). The samples were mixed thoroughly and incubated in the dark for 30 min at room temperature (RT). Then, erythrocyte lysis was performed by adding 2 mL of lysing solution without paraformaldehyde (Pharm lyse Solution, BD Bioscience, San Jose, CA, USA) and 0.5 mL of lysing solution with formaldehyde (FACS Lysing Solution, BD Bioscience) diluted according to the manufacturer’s instructions. After mixing, the samples were incubated for 5 min at room temperature (RT) in the dark. After incubation, the samples were washed twice with 2 mL of phosphate-buffered saline (PBS) enriched with 5% FBS (Stain Buffer FBS, BD Pharmingen), centrifuged (5 min, 300× *g*), and the supernatant was poured off. After the last centrifugation, the obtained cell pellet was suspended in 300 μL of PBS. Samples were analyzed immediately after the staining procedure. Ultimately, 5000 cells were collected in the monocytic gate (SSCmed/CD45high). Cell analysis consisted of multi-stage gating (Figure 1). In the first step, leukocytes were gated according to the size (Forward Scatter—FSC) and granularity (SSC) of cells. Damaged cells and cell clumps (doubles) were excluded from the analysis. Then, the neutrophil, monocyte, and lymphocyte populations were gated based on SSC/CD45 parameters. The percentage of cells expressing the membrane IGF-1R and its density on the cells (MFI—median fluorescence intensity of the fluorochrome used to label the receptor) were analyzed in the leukocyte populations. The gating strategy is shown in Figure 1. Isotype controls were included in each study panel to determine receptor-positive cells. Data analysis was performed using FlowJo 7.5.5 (Tree Star, Inc., Ashland, OR, USA).

### 2.7. Statistical Analysis

The normal distribution of the analyzed variables was tested with Shapiro–Wilk test. The results obtained for most of the introduced variables were statistically significant, meaning their distributions differ significantly from the normal distribution. Therefore, it was justified to conduct further analyses based on non-parametric tests. Differences between both groups were tested using the Mann–Whitney U test (with continuity correction). Variables were presented as median and range of quartile values (lower and upper). The relationships between the studied parameters were determined using Spearman’s rank-order correlation. Statistically significant results were estimated at *p* < 0.05. All statistical analyses were performed using the TIBCO Statistica version 13.3 program (TIBCO Software Inc., Santa Clara, CA, USA).

## 3. Results

### 3.1. Characteristics of the Study Groups

Both groups differed in cardiovascular parameters (except for the relative thickness of the left ventricle walls) and BMI values: the PH patients had significantly greater BMI values in comparison with the normotensive control group (*p* = 0.000021). The values of biochemical parameters did not differ between the groups, except for an increase in triglyceride levels in adolescents with PH. The anthropometric and clinical characteristics of both groups are presented in Table 1.

### 3.2. Gene Expression Profile: IGF-1 and IGF-1 Receptor (IGF-1R) and IGF-1 Plasma Concentration in Pediatric Patients with Primary Hypertension (PH) and Healthy Children

The PH patients had significantly reduced leukocyte IGF-1R gene expression (0.78 [0.2–17.6]) as compared to the control group (1.03 [0.83–1.17]; *p* = 0.0017), with no changes in the expression of the leukocyte IGF-1 gene (*p* = 0.92).

IGF-1 plasma concentration tended to be increased in the PH children but without statistical significance, although the value was close to *p* < 0.05 (420 [388.5–457.8] vs. 369.6 [333.9–443.1]; *p* = 0.07). The results in both groups were within the reference range for age and gender [16].

Furthermore, statistically significant negative correlations were found between the IGF-1 expression and HMOD parameters, including PWV (r = −0.49), SBP (r = −0.44), cIMT (r = −0.43), and between the IGF-1R expression and PWV (r = −0.42) and SBP (r = −0.41).

### 3.3. The IGF-1 Receptor (CD221) Expression in the Subpopulation of Lymphocytes, Monocytes, and Neutrophils in the PH Children/Adolescents and Healthy Controls

**Figure 2:**
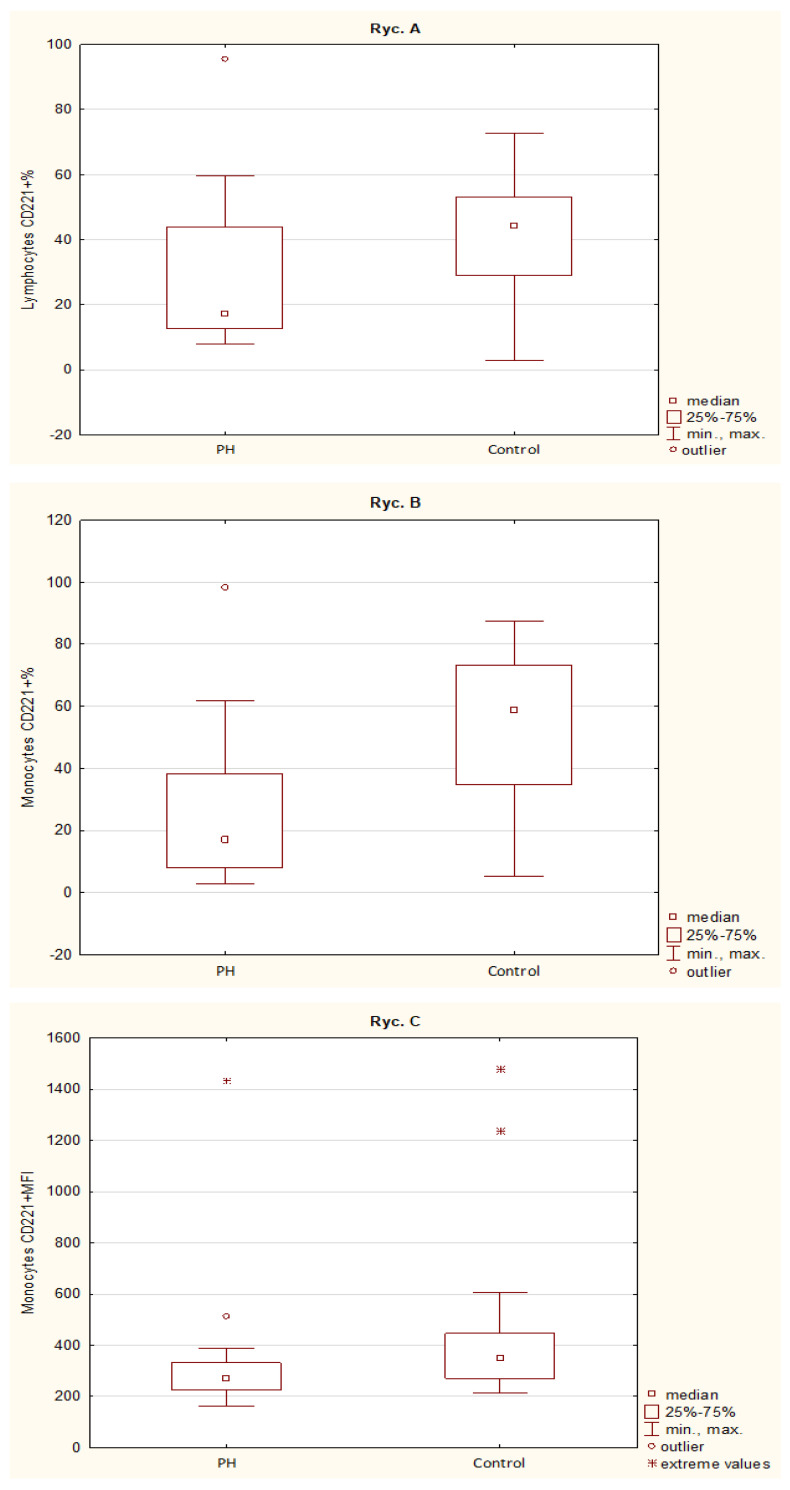
Expression of the IGF-1 receptor (CD221) on leukocyte subpopulations. The percentage of lymphocytes (**A**), monocytes (**B**) expressing the IGF-1 receptor (CD221%), and the density of the CD221 receptor (MFI) on monocytes (**C**) are presented (*p*-value < 0.05—statistically significant differences). PH—children with primary hypertension; Control—control group.

The data are presented in terms of the percentage of positive cells [%] and receptor density (MFI) in the tested cell subsets. The PH children had decreased percentage of lymphocytes (17.3 [12.6–44] vs. 44.3 [28.9–53], *p* = 0.02) and monocytes (16.8 [7.94–38.3] vs. 58.9 [34.8–73.1], *p* = 0.0003) bearing IGF-1R receptor (CD221), as well as lower MFI for IGF-1R on monocytes (271 [224–331] vs. 349 [271–446], *p* = 0.022) [Figure 2] but not on lymphocytes (*p* = 0.15) in compare to control group. Neutrophils did not show significant changes in the IGF-1R expression pattern.

## 4. Discussion

The assumptions of the study are cognitive. There are few reports of studies in adults, but they have many aggravating factors (smoking, stimulants, bad eating habits, previous diseases, etc.), which make the interpretation of the results difficult. Research conducted in a group of children and adolescents with PH minimizes the consequences related to lifestyle. Studying the mechanisms of development of primary hypertension is an important medical aspect, as there is an increasing tendency in the incidence of this disease, especially among children during puberty. If left untreated, it may lead to organ damage—heart, vascular endothelium, and kidneys.

The present study showed that PH children had unchanged IGF-1 plasma concentration and leukocyte IGF-1 expression but a decreased population of IGF-1R-bearing lymphocytes and monocytes and low IGF1-R density in monocytes. The IGF-1 expression was negatively correlated with PWV, SP, and cIMT. IGF-1R expression negatively correlated with PWV and SBP.

This is the first report on the expression of the IGF-1/IGF-1R axis in PH children/adolescents. Many publications underline the close relationship between IGF-1 production and age/gender [17]. PH is usually diagnosed during puberty. Hence, we adopted the general age group as the age of puberty, without division by gender. The measured IGF-1 concentration was within the average age range for children recruited for the study [16].

Many studies indicate decreased serum IGF-1 concentration in people with PH [18]. The IGF-1 has strong vasorelaxant properties and increases IGF-1 signaling in hypertension [19]. Increased circulating IGF-1 in plasma reduces the risk of hypertension in women aged 30–55 [19]. Low serum IGF-1 levels predict the development of early cardiovascular disorders and hypertension [20]. The above data were based on experimental models or studies in adults.

We found here that the PH children had unchanged plasma and leukocyte IGF-1 expression, which means that both systemic and local IGF-1 production is unchanged. This may suggest that IGF-1 levels in PH children are continuously maintained to ensure anti-inflammatory and protective effects in the early stage of PH development (children/adolescents).

The level of IGF-1 is regulated by many factors (insulin resistance, insulin levels, lipids, vitamin D3, hormones, metabolic syndrome, and inflammatory parameters) [20,21,22,23,24]. IGF-1 concentration in the upper reference range is associated with reduced blood pressure and vascular tone [25]. The regulation of circulating IGF-1 levels is modified by binding proteins, i.e., IGFBPs, especially IGFBP3 [25]. Compensatory mechanisms function during puberty. It is also characterized by faster bone turnover and generally faster growth and weight gain in adolescence with PH [26]. High growth hormone levels exert pressure by increasing plasma volume and sodium retention [18]. Moreover, in young people with hypertension, faster biological development has also been observed, characterized by the predominance of the catabolic process, including a reduction in the level of IGF-1 [27].

Our results show a negative correlation of leukocyte IGF-1 expression with cardiovascular parameters, i.e., PWV, cIMT, and SBP, confirming this protein’s protective role. Higher plasma IGF-1 levels lower blood pressure because they dilate blood vessels by influencing NO synthesis [18]. These studies are consistent with other reports in adults, women, and men aged 30–65 [28], in type I diabetes [22], in people with hypertension and type I diabetes [25], and in women [19] or men [29]. A relationship between the IGF-1 plasma concentration and the elastin level was demonstrated in infants with intrauterine hypotrophy. Low levels of IGF are decreased in elastase synthesis, which increases arterial stiffness and contributes to the development of hypertension [30].

IGF-1 is a strong mitogenic and anti-apoptotic factor [31] and has antioxidant, anti-fibrotic, and repair properties for the vascular endothelium [24]. Our study did not assess the IGF-1 bioavailability index because we did not assess IGF-1 binding protein concentration in plasma (IGFBP3). Chronic, low-grade, systemic inflammation that characterizes primary hypertension may influence changes in the expression level of IGFBP-binding proteins, which may limit the bioavailability of IGF-1, change the IGF-1/IGF-1R signaling, and IGF-1R receptor resistance to IGF-1 binding. These changes may also be caused by epigenetic regulation by the action of small endogenous, non-coding RNAs, the so-called miRNAs [32]. They have an essential function during inflammation, post-transcriptionally modulating the expression of various genes [33,34].

We found reduced IGF-1R expression in leukocytes in children with PH compared to normotensive controls.

The level of leukocyte IGF-1R expression is controlled by serum IGF-1 concentration [35]. The decrease in serum IGF-1 levels is associated with increased leukocyte IGF-R expression and low IRS1 and IRS2 expression. On the contrary, the elevation of IGF-1 serum levels results in the downregulation of IGF-R expression and elevation of mRNA expression of the adapter proteins IRS1 and IRS2 [35,36].

In children with PH, plasma IGF-1 levels did not differ from normotensive controls. A local, compensatory increase in IGF-1 expression in leukocytes or increased IGF-1/IGF-1R signaling in plasma contributes to the decrease in leukocyte IGF-1R expression in PH children.

The IGF-1R depends on the activation status and cell type [37,38]. The highest IGF-1R expression has been found in cardiomyocytes, smooth muscle cells, and vascular endothelial cells, as well as cells of the immune system, i.e., T and B lymphocytes, monocytes, dendritic cells, granulocytes, and NK cells [23].

The results presented here indicate that the PH children had a reduced percentage of lymphocytes and monocytes expressing the IGF-1R (CD221). Moreover, the PH children’s monocytes had low IGF-1R density. The reduction in IGF-1R expression in the lymphocyte and monocyte population may depend on the mechanism described above that decreases IGF-1R expression via the action of IGF-1. The second possibility may depend on accelerated biological aging of the immune system (“premature senescence”) described in primary arterial hypertension. This process is associated with chronic induction of the immune system by pro-inflammatory factors [37]. A decrease in IGF-1 receptor expression impairs vascular endothelial function, increases the infiltration of inflammatory cells, and worsens fibrotic renal disease [38]. The physiological level of IGF-1R expression, maintained in pro- and anti-inflammatory response balance, determines the optimal response to IGF-1 stimulation.

Metalloproteinases may also regulate IGF-1R expression. Mouse model studies (spontaneous, essential hypertension) have shown that the proteolytic effect of extracellular matrix metalloproteinases causes the degradation of the alpha subunit of the IGF-1R receptor of cells in various tissue areas (peripheral blood leukocytes, aorta, kidneys, liver, and spleen). This resulted in the absence or reduction in response to the IGF-1 hormone (IGF-1 resistance), which means a reduction in intracellular signaling and dysfunction of the vascular endothelium and an increase in vascular resistance [11]. This mechanism may also occur in children with PH, who showed increased expression of the gene for metalloproteinase-2 (MMP-2) in leukocytes [39].

Our study has some limits. IGF-1 level depends on gender and age. Because of the recruitment of a small group for the study, we did not divide it according to that (male gender predominance). The IGF-1 hormone is also challenging to interpret because it may vary among individual factors. It depends on various interfering factors: BMI, nutrition, walking speed, and hormonal regulation. The regulation of the level and function of IGF-1 is very complex and is controlled by the growth hormone (GH), the concentration of which decreases with age. It also depends on genetic, environmental (e.g., diet), and endocrine (e.g., insulin and glucocorticoids) factors. The reference range presented during puberty is broad and depends on many factors. The concentration of IGF-1 also fluctuates more frequently but is generally at a high level. However, the level of circulating IGF-1 in plasma, considered individually for each in our study, was within the normal range appropriate for age and sex. With age, this level decreases, which reduces the elasticity of the endothelium of blood vessels. In women, it is usually lower. However, these gender differences are apparent in adults and older people. At adolescence, the concentration of IGF-1 reaches its highest values [40].

## 5. Conclusions

We conclude that the IGF-1/IGF-1R axis in the PH children possibly preserves their anti-inflammatory and protective action, showing significant negative correlations with parameters of HMOD: PWV, SP, and cIMT. The mechanisms responsible for this process still need to be thoroughly understood.

Moreover, strong cell renewal processes prevail in pubertal children, which indicates the activation of protective proteins and signaling pathways [23,24,40].

The current research can also be extended to analyze additional molecules that may influence the regulation of the mechanism of hypertension.

## Figures and Tables

**Table 1:** Characteristics of the study groups (patients and children from the control group). Median values are given for parameters with quartiles in brackets. The *p*-value < 0.05 (statistically significant differences) is marked in bold.

Parameters	PH/-Control	Median and Quartiles PH Patients	Median and Quartiles Control Group	Significance Level (*p* < 0.05—Statistically Significant Differences)
Age	39/35	15.93 (14.9–17.2)	15.2 (14.4–16.7)	0.14
BMI (kg/m^2^)	37/31	25.66 (22.8–28.18)	21.22 (18.34–23.34)	**0.000021**
Total Cholesterol (mg/dL)	35/35	155 (140–183)	156 (144–173)	0.90
LDL (mg/dL)	35/35	85 (76–104)	89 (75–116)	0.52
HDL (mg/dL)	35/35	48 (42–58)	48 (41–59)	0.76
TG (mg/dL)	35/35	85 (63–132)	63 (48–98)	**0.009**
Glucose (mg/dL)	36/35	88 (84–92.8)	88 (85–91)	0.97
Insulin (μIU/mL)	33/35	12.3 (10.4–15.6)	12.3 (7.02–15.9)	0.33
AoBP (mm Hg)	28/30	121 (116.92–123.63)	108 (100–114.7)	**0.000001**
SBP (mm Hg)	28/30	134.33 (128–140.88)	112 (106–118)	**0.000000**
DBP (mm Hg)	28/30	70 (66.96–75.83)	66 (63–70)	**0.0112**
PWV (m/s)	28/30	6.09 (5.69–6.81)	5.5 (5–5.7)	**0.0004**
cIMT (mm)	33/31	0.44 (0.44–0.47)	0.42 (0.41–0.43)	**0.000005**
WCSA (mm^2^)	30/30	7.28 (6.97–7.62)	6.63 (6.2–7.34)	**0.005**
LVMi (g/m^2^)	32/28	34.77 (31–41)	29.99 (26.81–32.96)	**0.003**
RWT (mm)	32/28	0.37 (0.35–0.42)	0.36 (0.33–0.47)	0.59

Abbreviations: BMI—body mass index; LDL—low-density lipoprotein; HDL—high-density lipoprotein; TG—triglycerides; AoBP—aortic blood pressure; SBP—systolic blood pressure; DBP—diastolic blood pressure; PWV—pulse-wave velocity; cIMT—carotid intima-media thickness; WCSA—wall cross-sectional area; LVMi—left ventricular mass index; RWT—relative wall thickness.

## Data Availability

The data are available upon request.
